# Supplementary material for: High-purity AAV vector production utilizing recombination-dependent minicircle formation and genetic coupling
Source: EMBO Mol Med. 2025 May 16;17(6):1475–94. doi: 10.1038/s44321-025-00248-w (PMC12162853; doi:10.1038/s44321-025-00248-w)
Supplement: Supplementary file 1 — Table EV1 [file 44321_2025_248_MOESM1_ESM.pdf]

Table EV1. Relative plasmid mass usage in standard triple transfection and AAVPure[Mfg].

	pHelper (15.4 kb)	pTrans (8.0 kb)	pCis (5.1 kb)	pHelper_ DBP-2A-Bxb1 (17.0 kb)	pHelper_ DBP-IRES-Bxb1 (17.6 kb)	pTrans/Cis (10.4 kb)	pCAG.Bxb1 (6.4 kb)
Triple transfection	1	1	1				
AAVPure[Mfg] 1.0				1.1		1.3	
AAVPure[Mfg] 1.1					1.1	1.3	
AAVPure[Mfg] 2.0	1			0.01		1.3	
AAVPure[Mfg] 2.1	1					1.3	0.01
AAVPure[Mfg] 3.0	1					1.3	
