# Supplementary material for: High-purity AAV vector production utilizing recombination-dependent minicircle formation and genetic coupling
Source: EMBO Mol Med. 2025 May 16;17(6):1475–94. doi: 10.1038/s44321-025-00248-w (PMC12162853; doi:10.1038/s44321-025-00248-w)
Supplement: Supplementary file 2 — Table EV2 [file 44321_2025_248_MOESM2_ESM.pdf]

Table EV2. Vector DNA impurities characterized by PacBio sequencing.

**Read counts**	**Triple Transfection**	**AAVPure^{Mfg} 1.0**
Total	688,729 (100%)	538,271 (100%)
Transgene	577,152 (83.8%)	473,339 (87.94%)
Plasmid backbone	12,372 (1.80%)	330 (0.06%)
Host cell DNA	33,825 (4.91%)	22,673 (4.21%)
Helper genes	14,425 (2.09%)	10,466 (1.94%)
rcAAV (ITR-flanked Rep-Cap)	0	0
Rep-Cap (without ITR)	1	1
ITR-Rep	61 (0.009%)	246 (0.046%)
Cap-ITR	696 (0.101%)	302 (0.056%)

rcAAV: replication-competent AAV, ITR: inverted terminal repeat
