# Supplementary material for: High-purity AAV vector production utilizing recombination-dependent minicircle formation and genetic coupling
Source: EMBO Mol Med. 2025 May 16;17(6):1475–94. doi: 10.1038/s44321-025-00248-w (PMC12162853; doi:10.1038/s44321-025-00248-w)
Supplement: Supplementary file 3 — Table EV3 [file 44321_2025_248_MOESM3_ESM.pdf]

Table EV3. Plasmid sequences

<table>
<tr><th>Plasmid</th><th>DNA Sequence</th></tr>
<tr><td>pRep2/Cap2</td><td>Region between P40 and Intron in pTrans of pRep2/Cap2 packaging plasmid</td></tr>
<tr><td colspan="2">gttgcgcagccatcgacgtcagacgcggaagcttcgatcaactacgcagacag</td></tr>
<tr><td>pRep2-attR1/Cap2 (pHL280b)</td><td>attR insertion in site 1 (attR1) between P40 and Intron (Spacer)
Note: Bxb1-Ga mutant attR site was used in this study[28].</td></tr>
<tr><td colspan="2">gttGTGGTTTGTCTGGTCAACCACCGCGGaCTCCGTCGTCAGGATCATtcagttgcgcagccatcgacgtcagacgcggaagcttcgatcaactacgcagacag</td></tr>
<tr><td>pRep2-attR2/Cap2 (pHL281b)</td><td>attR insertion in site 2 (attR2) between P40 and Intron (best performer, used in following experiments) (Spacer)</td></tr>
<tr><td colspan="2">gttgcgcagccatcgacgtcaGTGGTTTGTCTGGTCAACCACCGCGGaCTCCGTCGTCAGGATCATtcagacgcggaagcttcgatcaactacgcagacag</td></tr>
<tr><td>pRep2-attR3/Cap2 (pHL282b)</td><td>attR insertion in site 3 (attR3) between P40 and Intron</td></tr>
<tr><td colspan="2">gttgcgcagccatcgacgtcagacgcggaagcttcgGTGGTTTGTCTGGTCAACCACCGCGGaCTCCGTCGTCAGGATCATatcaactacgcagacag</td></tr>
<tr><td>pHelper_DBP-2A-Bxb1 (pHL284)</td><td>DBP (20bp)-T2A-Bxb1-DBP 3’UTR (20bp)</td></tr>
<tr><td colspan="2">ggcagaaccccttgattttGGATCCGGTGAGGGCAGAGGAAGTCTACTAACATGCGGTGACGTGGAGGAGAATCCGGGCCCTatgccaaaaaagaaaagaaaagtgtatccctatgatgtccccgattatgccggttcaagagccctggtcgtgattagactgagccgagtgacagacgccaccacaagtcccgagagacagctggaatcatgccagcagctctgtgctcagcggggttgggatgtggtcggcgtggcagaggatctggacgtgagcggggccgtcgatccattcgacagaaagaggaggcccaacctggcaagatggctcgctttcgaggaacagccctttgatgtgatcgtcgcctacagagtggaccggctgacccgctcaattcgacatctccagcagctggtgcattgggctgaggaccacaagaaactggtggtcagcgcaacagaagcccacttcgatactaccacaccttttgccgctgtggtcatcgcactgatgggcactgtggcccagatggagctcgaagctatcaaggagcgaaacaggagcgcagcccatttcaatattagggccggtaaatacagaggctccctgccccccttggggatatctccctaccagggtggatggggagtggagactggtgccagaccccgtccagagagagcggattctggaagtgtaccacagagtggtcgataaccacgaaccactccatctggtggcacacgacctgaatagacgcggcgtgctctctccaaaggattattttgctcagctgcagggaagagagccacagggaagagaatggagtgctactgcactgaagagatctatgatcagtgaggctatgctgggttacgcaacactcaatggcaaaactgtccgggacgatgacggagcccctctggtgagggctgagcctattctcaccagagagcagctcgaagctctgcgggcagaactggtcaagactagtcgcgccaaacctgccgtgagcaccccaagcctgctcctgagggtgctgttctgcgccgtctgtggagagccagcatacaagtttgccggcggagggcgcaaacatccccgctatcgatgcaggagcatggggttccctaagcactgtggaaacgggacagtggccatggctgagtgggacgccttttgcgaggaacaggtgctggatctcctgggtgacgctgagcggctggaaaaagtgtgggtggcaggatctgactccgctgtggagctggcagaagtcaatgccgagctcgtggatctgacttccctcatcggatctcctgcatatagagctgggtccccacagagagaagctctggacgcacgaattgctgcactcgctgctagacaggaggaactggagggcctggaggccaggccctctggatgggagtggcgagaaaccggacagaggtttggggattggtggagggagcaggacaccgcagccaagaacacatggctgagatccatgaatgtccggctcacattcgacgtgcgcggtggcctgactcgaaccatcgattttggcgacctgcaggagtatgaacagcacctgagactggggtccgtggtcgaaagactgcacactgggatgtcctaaacccttgccgtctgcgccgt</td></tr>
<tr><td>pTrans-STOP (pHL327)</td><td>attP-3XSV40 pA-attB insertion in site 2 between P40 and Intron</td></tr>
<tr><td colspan="2">gttgcgcagccatcgacgtcaGTGGTTTGTCTGGTCAACCACCGCGGaCTCAGTGGTGTACGGTACAAACCCAttaattaaggccttaattaggctgcgcgaagcttgcagatctgcgactctagaggatctgcgactctagaggatcataatcagccataccacatttgtagaggttttacttgctttaaaaaacctcccacacctccccctgaacctgaaacataaaatgaatgcaattgttgttgttaacttgtttattgcagcttataatggttacaaataaagcaatagcatcacaaatttcacaaataaagcatttttttcactgcattctagttgtggtttgtccaaactcatcaatgtatcttatcatgtctggatctgcgactctagaggatcataatcagccataccacatttgtagaggttttacttgctttaaaaaacctcccacacctccccctgaacctgaaacataaaatgaatgcaattgttgttgttaacttgtttattgcagcttataatggttacaaataaagcaatagcatcacaaatttcacaaataaagcatttttttcactgcattctagttgtggtttgtccaaactcatcaatgtatcttatcatgtctggatctgcgactctagaggatcataatcagccataccacatttgtagaggttttacttgctttaaaaaacctcccacacctccccctgaacctgaaacataaaatgaatgcaattgttgttgttaacttgtttattgcagcttataatggttacaaataaagcaatagcatcacaaatttcacaaataaagcatttttttcactgcattctagttgtggtttgtccaaactcatcaatgtatcttatcatgtctggatcccgagcgcgcagccttaattaaGGCTTGTCGACGACGGCGGaCTCCGTCGTCAGGATCATtcagacgcggaagcttcgatcaactacgcggacag</td></tr>
</table>

Table EV3. Plasmid sequences

**Plasmid**	**DNA Sequence**
pHelper_DBP-IRES-Bxb1 (pHL285)	DBP (20bp)-IRES-Bxb1-DBP 3’UTR (20bp) (*Spacer*)
agaacccctttgatttttaa*TGCAT*cccctctccctcccccccccctaacgttactggccgaagccgcttggaataaggccggtgtgcgtttgtctatatgttatttt<br>ccaccatattgccgtcttttggcaatgtgagggcccggaaacctggccctgtcttcttgacgagcattcctaggggtctttcccctctcgccaaaggaatgcaagg<br>tctgttgaatgtcgtgaaggaagcagttcctctggaagcttcttgaagacaaacaacgtctgtagcgaccctttgcaggcagcggaaccccccacctggcgac<br>aggtgcctctgcggccaaaagccacgtgtataagatacacctgcaaaggcggcacaaccccagtgccacgttgtgagttggatagttgtggaaagagtcaa<br>atggctctcctcaagcgtattcaacaaggggctgaaggatgcccagaaggtaccccattgtatgggatctgatctggggcctcggtacacatgctttacatgtgtt<br>tagtcgaggttaaaaaaacgtctaggccccccgaaccacggggacgtggttttcctttgaaaaacacgatgataatatggccacaaccatgccaaaaaaga<br>aaagaaaagtgtatccctatgatgtccccgattatgccggttcaagagccctggtcgtgattagactgagccgagtgacagacgccaccacaagtcccgaga<br>gacagctggaatcatgccagcagctctgtgctcagcggggttgggatgtggtcggcgtggcagaggatctggacgtgagcggggccgtcgatccattcgaca<br>gaaagaggaggcccaacctggcaagatggctcgctttcgaggaacagccctttgatgtgatcgtcgcctacagagtggaccggctgacccgctcaattcga<br>catctccagcagctggtgcattgggctgaggaccacaagaaactggtggtcagcgcaacagaagcccacttcgatactaccacaccttttgccgctgtggtca<br>tcgcactgatgggcactgtggcccagatggagctcgaagctatcaaggagcgaaacaggagcgcagcccatttcaatattagggccggtaaatacagagg<br>ctccctgccccccttggggatatctccctaccagggtggatggggagtggagactggtgccagaccccgtccagagagagcggattctggaagtgtaccaca<br>gagtggtcgataaccacgaaccactccatctggtggcacacgacctgaatagacgcggcgtgctctctccaaaggattattttgctcagctgcagggaagag<br>agccacagggaagagaatggagtgctactgcactgaagagatctatgatcagtgaggctatgctgggttacgcaacactcaatggcaaaactgtccgggac<br>gatgacggagcccctctggtgagggctgagcctattctcaccagagagcagctcgaagctctgcgggcagaactggtcaagactagtcgcgccaaacctgc<br>cgtgagcaccccaagcctgctcctgagggtgctgttctgcgccgtctgtggagagccagcatacaagtttgccggcggagggcgcaaacatccccgctatcg<br>atgcaggagcatggggttccctaagcactgtggaaacgggacagtggccatggctgagtgggacgccttttgcgaggaacaggtgctggatctcctgggtga<br>cgctgagcggctggaaaaagtgtgggtggcaggatctgactccgctgtggagctggcagaagtcaatgccgagctcgtggatctgacttccctcatcggatct<br>cctgcatatagagctgggtccccacagagagaagctctggacgcacgaattgctgcactcgctgctagacaggaggaactggagggcctggaggccagg<br>ccctctggatgggagtggcgagaaaccggacagaggtttggggattggtggagggagcaggacaccgcagccaagaacacatggctgagatccatgaat<br>gtccggctcacattcgacgtgcgcggtggcctgactcgaaccatcgattttggcgacctgcaggagtatgaacagcacctgagactggggtccgtggtcgaa<br>agactgcacactgggatgtcctaaacccttgccgtctgcgccgt	
pTrans/Cis.Rep2-EGFP-Cap2 (pHL300)	attP-5’ ITR-CB6-EGFP-pA-3’ ITR-attB insertion in site 2 between P40 and Intron (*Spacer*)<br>Note: Bxb1-Ga mutant attP and attB target sites were used in this study[28].
gttgcgcagccatcgacgtcaGTGGTTTGTCTGGTCAACCACCGCGGaCTCAGTGGTGTACGGTACAAACCCA*ttaatta*<br>*aggccttaattagg*ctgcgcgctcgctcgctcactgaggccgcccgggcaaagcccgggcgtcgggcgacctttggtcgcccggcctcagtgagcgagcga<br>gcgcgcagagagggagtggccaactccatcactaggggttcct*tgtagttaatgattaacccgccatgctacttatctaccagggtaatggggatcctctagaa*<br>*ctatag*ctagtcgacattgattattgactagttattaatagtaatcaattacggggtcattagttcatagcccatatatggagttccgcgttacataacttacggtaaat<br>ggcccgcctggctgaccgcccaacgacccccgcccattgacgtcaataatgacgtatgttcccatagtaacgccaatagggactttccattgacgtcaatggg<br>tggagtatttacggtaaactgcccacttggcagtacatcaagtgtatcatatgccaagtacgccccctattgacgtcaatgacggtaaatggcccgcctggcatt<br>atgcccagtacatgaccttatgggactttcctacttggcagtacatctacgtattagtcatcgctattaccatgtcgaggccacgttctgcttcactctccccatctccc<br>ccccctccccacccccaattttgtatttatttattttttaattattttgtgcagcgatgggggcgggggggggggggcgcgcgccaggcggggcggggcggggcga<br>ggggcggggcggggcgaggcggagaggtgcggcggcagccaatcagagcggcgcgctccgaaagtttccttttatggcgaggcggcggcggcggcgg<br>ccctataaaaagcgaagcgcgcggcggg*cgggagcaagctttattgcggtagtttatcacagttaaattgctaacgcagtcagtgcttctgacacaacagtctc*<br>*gaacttaagctgcagaagttggtcgtgaggcactgggcaggtaagtatcaaggttacaagacaggtttaaggagaccaatagaaactgggcttgtcgagac*<br>*agagaagactcttgcgtttctgataggcacctattggtcttactgacatccactttgcctttctctccacaggtgtccactcccagttcaattacagctcttaaggctag*<br>*agtacttaatacgactcactataggctagtaatacgactcactatag*atggtgagcaagggcgaggagctgttcaccggggtggtgcccatcctggtcgagct<br>ggacggcgacgtaaacggccacaagttcagcgtgtccggcgagggcgagggcgatgccacctacggcaagctgaccctgaagttcatctgcaccaccgg<br>caagctgcccgtgccctggcccaccctcgtgaccaccctgacctacggcgtgcagtgcttcagccgctaccccgaccacatgaagcagcacgacttcttcaa<br>gtccgccatgcccgaaggctacgtccaggagcgcaccatcttcttcaaggacgacggcaactacaagacccgcgccgaggtgaagttcgagggcgacac<br>cctggtgaaccgcatcgagctgaagggcatcgacttcaaggaggacggcaacatcctggggcacaagctggagtacaactacaacagccacaacgtcta<br>tatcatggccgacaagcagaagaacggcatcaaggtgaacttcaagatccgccacaacatcgaggacggcagcgtgcagctcgccgaccactaccagc<br>agaacacccccatcggcgacggccccgtgctgctgcccgacaaccactacctgagcacccagtccgccctgagcaaagaccccaacgagaagcgcgat<br>cacatggtcctgctggagttcgtgaccgccgccgggatcactctcggcatggacgagctgtacaagtaa*agcggccctagcgtttaaacgggccctctagact*<br>*cgaggacggggtgaactacgcctgaggatcc*gatctttttccctctgccaaaaattatggggacatcatgaagccccttgagcatctgacttctggctaataaag<br>gaaatttattttcattgcaatagtgtgttggaattttttgtgtctctcactcg*gaagcaattcgttgatctgaatttcgaccacccataatacccattaccctggtagataa*<br>*gtagcatggcgggttaatcattaactaca*aggaacccctagtgatggagttggccactccctctctgcgcgctcgctcgctcactgaggccgggcgaccaaag<br>gtcgcccgacgcccgggctttgcccgggcggcctcagtgagcgagcgagcgcgcag*ccttaattaa*GGCTTGTCGACGACGGCGGaCTC<br>CGTCGTCAGGATCATtcagacgcggaagcttcgatcaactacgcagacag	

Table EV3. Plasmid sequences

<table>
<tr><th>Plasmid</th><th>DNA Sequence</th></tr>
<tr><td>pAAVS1 LHA-SA-P2A-BxB1-T2A-PuroR-bGH pA-RHA (pHL380)</td><td>AAVS1 LHA-SA-P2A-BxB1-T2A-PuroR-bGH pA-RHA (*Spacer*)</td></tr>
<tr><td colspan="2">CTCTCTCCTGAGTCCGGACCACTTTGAGCTCTACTGGCTTCTGCGCCGCCTCTGGCCCACTGTTTCCCCTT
CCCAGGCAGGTCCTGCTTTCTCTGACCTGCATTCTCTCCCCTGGGCCTGTGCCGCTTTCTGTCTGCAGCTT
GTGGCCTGGGTCACCTCTACGGCTGGCCCAGATCCTTCCCTGCCGCCTCCTTCAGGTTCCGTCTTCCTCC
ACTCCCTCTTCCCCTTGCTCTCTGCTGTGTTGCTGCCCAAGGATGCTCTTTCCGGAGCACTTCCTTCTCGG
CGCTGCACCACGTGATGTCCTCTGAGCGGATCCTCCCCGTGTCTGGGTCCTCTCCGGGCATCTCTCCTCC
CTCACCCAACCCCATGCCGTCTTCACTCGCTGGGTTCCCTTTTCCTTCTCCTTCTGGGGCCTGTGCCATCT
CTCGTTTCTTAGGATGGCCTTCTCCGACGGATGTCTCCCTTGCGTCCCGCCTCCCCTTCTTGTAGGCCTGC
ATCATCACCGTTTTTCTGGACAACCCCAAAGTACCCCGTCTCCCTGGCTTTAGCCACCTCTCCATCCTCTT
GCTTTCTTTGCCTGGACACCCCGTTCTCCTGTGGATTCGGGTCACCTCTCACTCCTTTCATTTGGGCAGCT
CCCCTACCCCCCTTACCTCTCTAGTCTGTGCTAGCTCTTCCAGCCCCCTGTCATGGCATCTTCCAGGGGTC
CGAGAGCTCAGCTAGTCTTCTTCCTCCAACCCGGGCCCCTATGTCCACTTCAGGACAGCATGTTTGCTGC
CTCCAGGGATCCTGTGTCCCCGAGCTGGGACCACCTTATATTCCCAGGGCCGGTTAATGTGGCTCTGGTT
CTGGGTACTTTTATCTGTCCCCTCCACCCCACA*ATCaagctt*ctgacctcttctcttcctcccacagg*gcctcgagagatctggcagcg*
*ga*gcaacaaacttctcactactcaaacaagcaggtgacgtggaggagaatcccgggcct*aggctcgag*atgccaaaaaagaaaagaaaagtgtatccct
atgatgtccccgattatgccggttcaagagccctggtcgtgattagactgagccgagtgacagacgccaccacaagtcccgagagacagctggaatcatgc
cagcagctctgtgctcagcggggttgggatgtggtcggcgtggcagaggatctggacgtgagcggggccgtcgatccattcgacagaaagaggaggccca
acctggcaagatggctcgctttcgaggaacagccctttgatgtgatcgtcgcctacagagtggaccggctgacccgctcaattcgacatctccagcagctggtg
cattgggctgaggaccacaagaaactggtggtcagcgcaacagaagcccacttcgatactaccacaccttttgccgctgtggtcatcgcactgatgggcactg
tggcccagatggagctcgaagctatcaaggagcgaaacaggagcgcagcccatttcaatattagggccggtaaatacagaggctccctgccccccttgggg
atatctccctaccagggtggatggggagtggagactggtgccagaccccgtccagagagagcggattctggaagtgtaccacagagtggtcgataaccacg
aaccactccatctggtggcacacgacctgaatagacgcggcgtgctctctccaaaggattattttgctcagctgcagggaagagagccacagggaagagaa
tggagtgctactgcactgaagagatctatgatcagtgaggctatgctgggttacgcaacactcaatggcaaaactgtccgggacgatgacggagcccctctgg
tgagggctgagcctattctcaccagagagcagctcgaagctctgcgggcagaactggtcaagactagtcgcgccaaacctgccgtgagcaccccaagcct
gctcctgagggtgctgttctgcgccgtctgtggagagccagcatacaagtttgccggcggagggcgcaaacatccccgctatcgatgcaggagcatggggtt
ccctaagcactgtggaaacgggacagtggccatggctgagtgggacgccttttgcgaggaacaggtgctggatctcctgggtgacgctgagcggctggaaa
aagtgtgggtggcaggatctgactccgctgtggagctggcagaagtcaatgccgagctcgtggatctgacttccctcatcggatctcctgcatatagagctgggt
ccccacagagagaagctctggacgcacgaattgctgcactcgctgctagacaggaggaactggagggcctggaggccaggccctctggatgggagtggc
gagaaaccggacagaggtttggggattggtggagggagcaggacaccgcagccaagaacacatggctgagatccatgaatgtccggctcacattcgacg
tgcgcggtggcctgactcgaaccatcgattttggcgacctgcaggagtatgaacagcacctgagactggggtccgtggtcgaaagactgcacactgggatgt
ccGGATCCGGTGAGGGCAGAGGAAGTCTACTAACATGCGGTGACGTGGAGGAGAATCCGGGCCCT
*GAATTCGCCACC*ATGACAGAATACAAGCCCACAGTCAGGCTTGCAACTAGAGATGACGTTCCCAGAGCAG
TGAGAACCCTGGCAGCTGCTTTTGCAGACTATCCGGCCACGAGGCACACTGTCGATCCCGATCGGCACAT
CGAGCGCGTTACAGAATTGCAGGAACTGTTCCTGACAAGAGTTGGGCTCGACATTGGTAAAGTGTGGGTC
GCTGACGACGGGGCAGCTGTTGCGGTGTGGACCACACCGGAGAGTGTGGAGGCCGGTGCTGTGTTTGCC
GAAATTGGTCCACGCATGGCCGAACTCTCCGGATCTCGGTTGGCCGCACAGCAGCAGATGGAAGGCCTG
CTGGCGCCTCACCGACCTAAAGAGCCTGCATGGTTTCTGGCCACCGTCGGCGTATCCCCCGATCATCAGG
GTAAGGGCCTCGGCAGCGCCGTCGTGCTGCCGGGTGTTGAGGCAGCTGAAAGAGCAGGCGTGCCGGCG
TTTTTGGAAACAAGTGCACCGAGGAATCTCCCATTTTACGAGAGACTGGGGTTCACCGTGACAGCCGATGT
CGAAGTGCCCGAAGGCCCCAGGACCTGGTGTATGACCCGCAAGCCCGGTGCCTAG*gatcc*gatctttttccctctgcc
aaaaattatggggacatcatgaagccccttgagcatctgacttctggctaataaaggaaatttattttcattgcaatagtgtgttggaattttttgtgtctctcactcg*T*
cccgatcccctatggtcgactctcagtacaatctgctctgatgccgcatagttaagccagtatctgctccctgcttgtgtgttggaggtcgctgagtagtgcgcgag
caaaatttaagctacaacaaggcaaggcttgaccgacaattgcatgaagaatctgcttagggttaggcgttttgcgctgcttcgcgatgtacgggccagatata
*cgcgt*GCCACTAGGGACAGGATTGGTGACAGAAAAGCCCCATCCTTAGGCCTCCTCCTTCCTAGTCTCCTGA
TATTGGGTCTAACCCCCACCTCCTGTTAGGCAGATTCCTTATCTGGTGACACACCCCCATTTCCTGGAGCC
ATCTCTCTCCTTGCCAGAACCTCTAAGGTTTGCTTACGATGGAGCCAGAGAGGATCCTGGGAGGGAGAGC
TTGGCAgggggtgggagggaagggggggaTGCGTGACCTGCCCGGTTCTCAGTGGCCACCCTGCGCTACCCTCTC
CCAGAACCTGAGCTGCTCTGACGCGGCCGTCTGGTGCGTTTCACTGATCCTGGTGCTGCAGCTTCCTTAC
ACTTCCCAAGAGGAGAAgcagtttggaaaaacaaaatcagaataagttggtcctgagttctaactttggctcttcacctttctagtccccaatttatattg
ttcctccgtgcgtcagttttacctgtgagataaggccagtagccagccccgtcctggcagggctgtggtgaggaGGGGGGTGTCCGTGTGGAAA
ACTCCCTTTGTGAGAATGGTGCGTCCTAGGTGTTCACCAGGTCGTGGCCGCCTCTACTCCCTTTCTCTTTC
TCCATCCTTCTTTCCTTAAAGAGTCCCCAGTGCTATCTGGGACATATTCCTCCGCCCAGAGCAGGGTCCCG
CTTCCCTAAGGCCCTGCTCTGGGCTTCTGGGTTTGAGTCCTTGGCAAGCCCAGGAGAGGCGCTCAGGCTT
CCCTGTCCCCCTTCCTCGTCCACCATCTCATGCCCCTGGCTCTCCTGCCCCTTCCCTACAGGGGTTCCTG
GCTCTGCTCTTCAGAC</td></tr>
</table>
