# Supplementary material for: High-purity AAV vector production utilizing recombination-dependent minicircle formation and genetic coupling
Source: EMBO Mol Med. 2025 May 16;17(6):1475–94. doi: 10.1038/s44321-025-00248-w (PMC12162853; doi:10.1038/s44321-025-00248-w)
Supplement: Supplementary file 4 — Appendix [file 44321_2025_248_MOESM4_ESM.pdf]

Appendix Figures

This file includes:

Appendix Figure S1 to S7

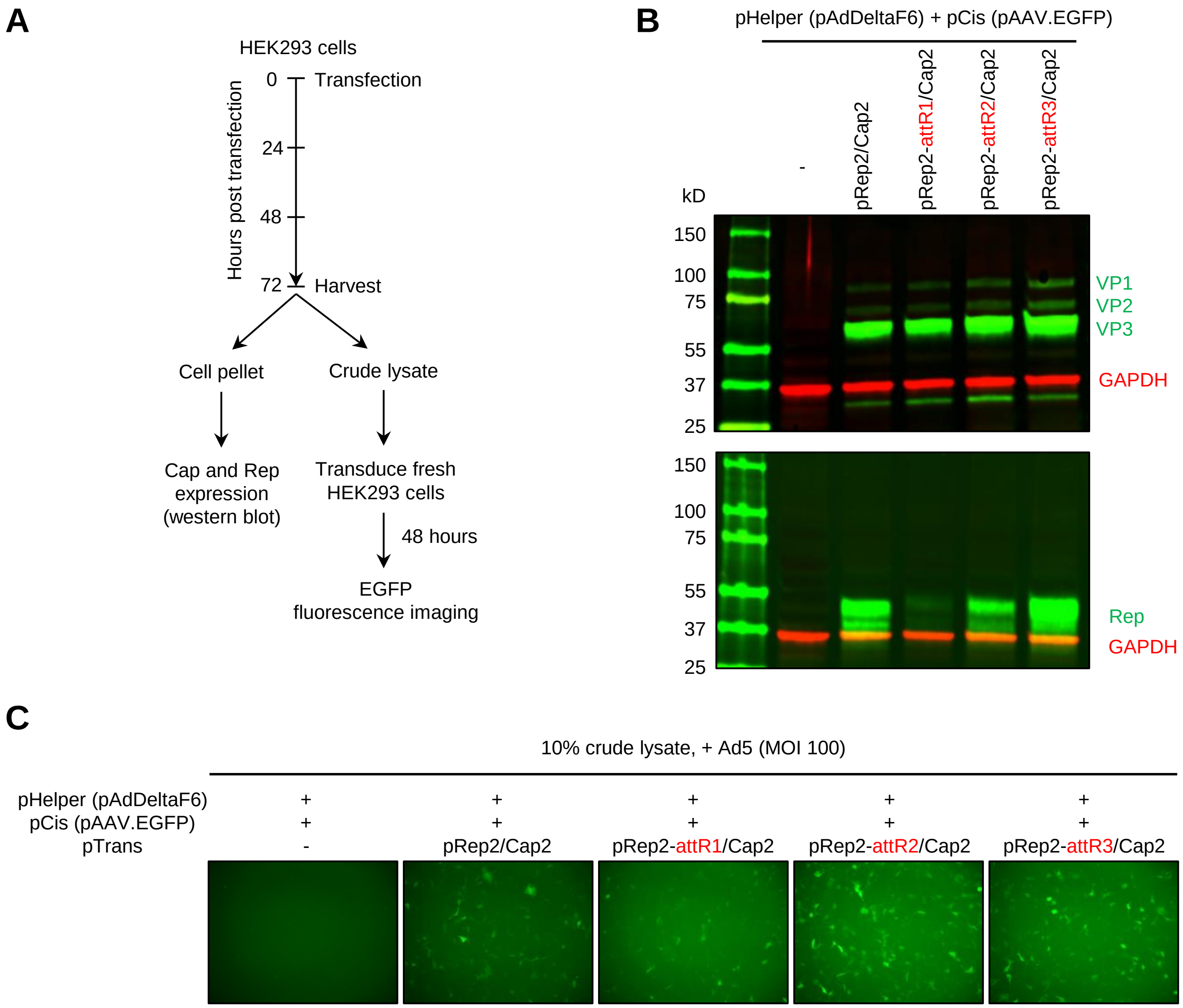

Appendix Figure S1. attR insertion in *Rep* does not impair Rep or Cap function.

A, Experimental procedure of Western blotting and AAV2.EGFP transduction assay. **B**, Western blotting of AAV2 VPs and Rep in triple transfection with conventional pRep2/Cap2 packaging plasmid or pRep2-attR/Cap2 variants. **C**, Representative fluorescence images of HEK293 cells infected with AAV2.EGFP-containing crude lysates. Equal volume (10%) of crude lysate in (B) was used to infect HEK293 cells in the presence of adenovirus 5 (Ad5). Images were taken 2 days post infection.

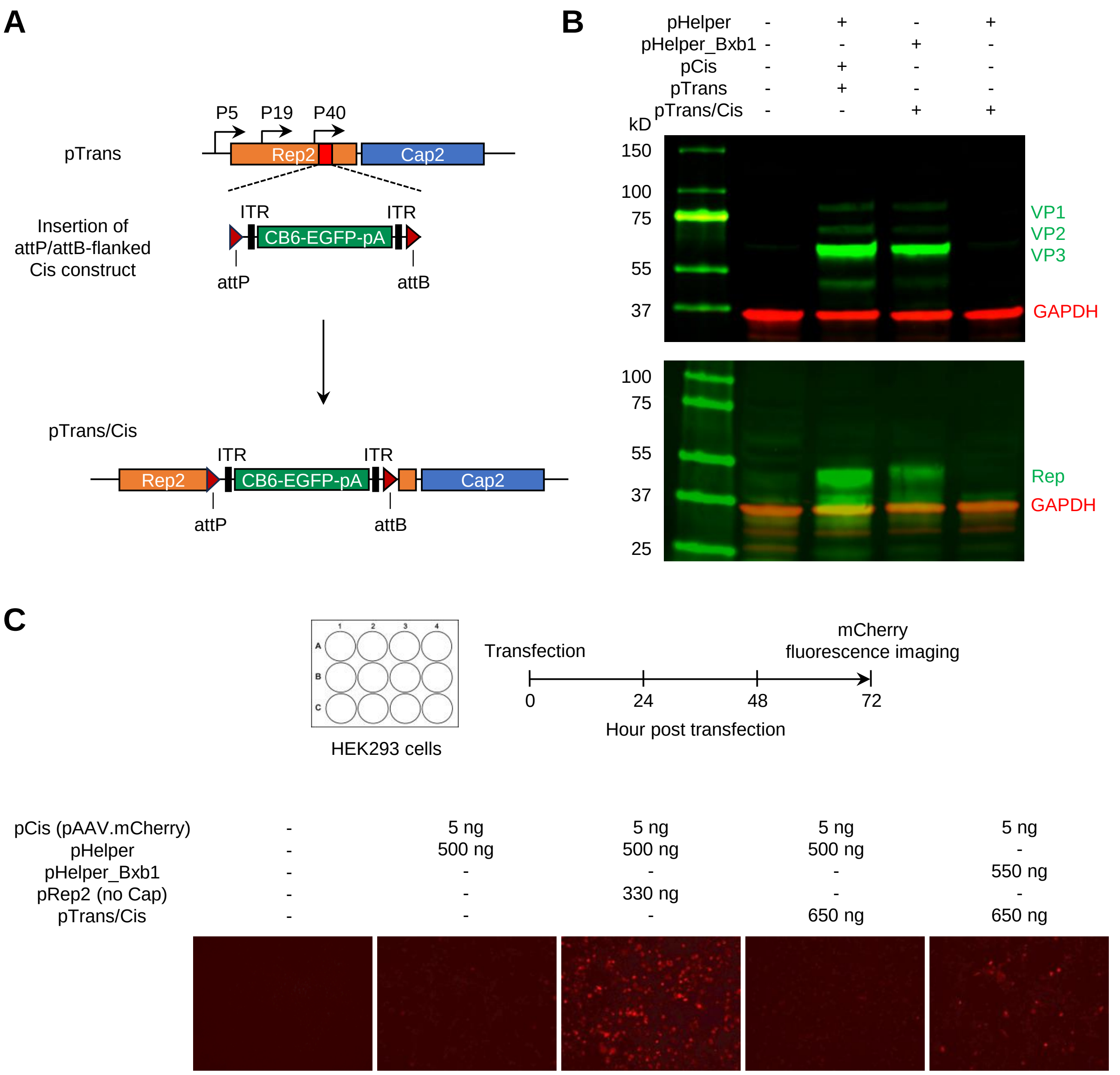

Appendix Figure S2. attP-ITR-Transgene-ITR-attB insertion in *Rep* abolishes Rep and Cap expression and function.

A, Schematic diagram illustrating the insertion of attP/attB-flanked Cis construct into the attR2 site in pRep2/Cap2 plasmid. **B**, Western blotting of AAV2 VPs and Rep in triple transfection or dual transfection with or without the *Bxb1* gene. **C**, Rep-dependent vector genome amplification assay. HEK293 cells were transfected with a small amount of pAAV.mCherry plasmid, along with other plasmids as indicated. Images were taken 3 days post transfection.

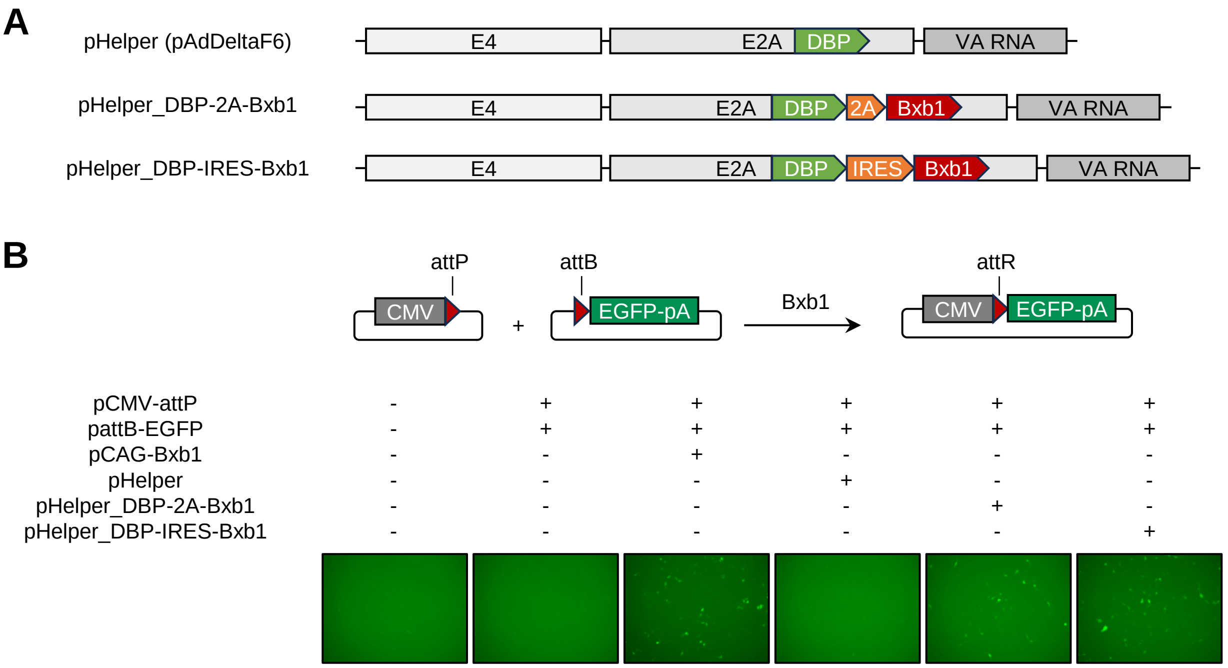

Appendix Figure S3. Design and validation of pHelper-Bxb1.

A, Schematics showing the plasmid components of the standard pHelper and modified versions carrying *Bxb1*. **B**, Reporter assay to test the Bxb1 recombination activity. HEK293 cells were transfected with pCMV-attP and pattB-EGFP, along with other plasmids as indicated. Images were taken 1 day post transfection.

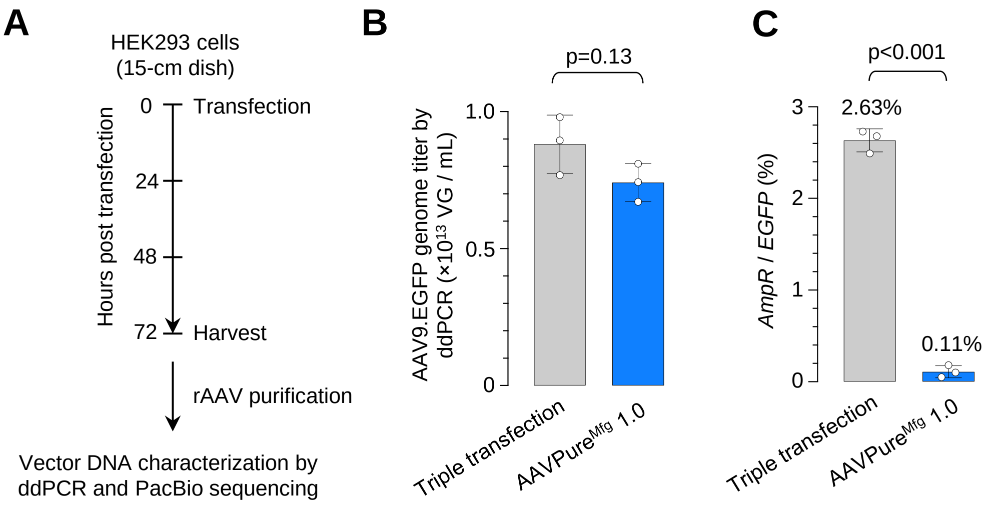

Appendix Figure S4. Characterization of the medium-scale AAV9.EGFP vectors by ddPCR.

A, Schematics showing the experimental workflow. **B**, Genome titers of AAV9.EGFP produced by either triple transfection or AAVPure^{Mfg} 1.0 in one 15-cm dish (medium scale). Cells were pelleted 72 h after transfection and purified by a commercial kit as described in Methods. Purified AAV vectors were treated with DNase-I and proteinase K, followed by ddPCR to determine titer. **C**, Plasmid backbone DNA levels in purified rAAV products by duplex ddPCR with one probe targeting the EGFP transgene and the other for AmpR. Data are mean ± s.d. of biological replicates. Statistical analysis was performed using unpaired t test.

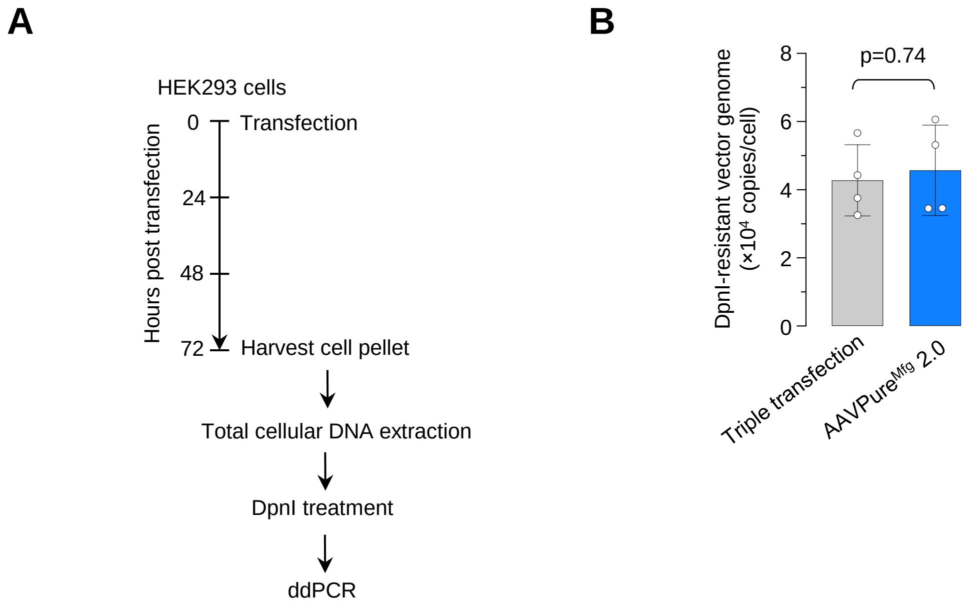

Appendix Figure S5. Comparable rescue and replication efficiency between conventional pCis and mcCis.

A, Schematics showing the experimental procedure. DpnI was used to digest residual plasmid DNA in purified total cellular DNA. **B**, Duplex ddPCR was performed with DpnI-treated cellular DNA, with one probe targeting *EGFP*, the other probe targeting *ACLY* as the normalization control. As four copies of *ACLY* are present in HEK293 nuclear genome, the *EGFP* copy number is normalized to 1/4 of the *ACLY* copy number to calculate the vector genome copies per cell. In (**B**), data are mean ± s.d. of biological replicates. Statistical analysis was performed using unpaired t test.

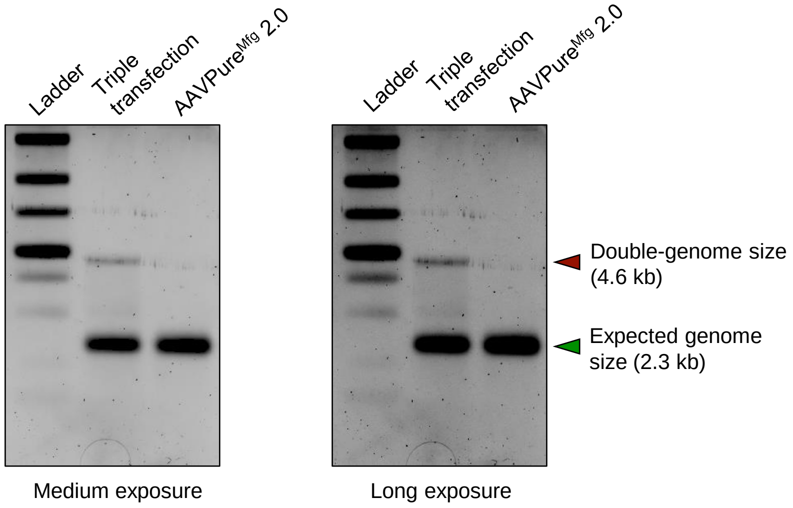

Appendix Figure S6. Alkaline gel images under high exposure conditions.

Denaturing alkaline gel images taken under medium, or long exposure time, showing AAV9.EGFP vector genome integrity. Green arrowhead indicates the full-length vector genome size; red arrowhead indicates double-genome size. The gel images taken under short exposure time was shown in Figure 5E.

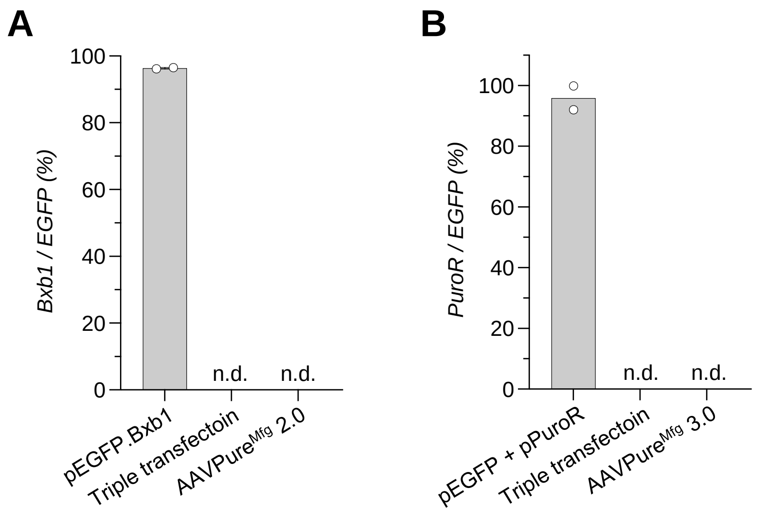

Appendix Figure S7. Potential *Bxb1* or *PuroR* gene contaminants in AAVPure^{Mfg} vectors were undetectable by ddPCR.

A, Duplex ddPCR was performed with vector DNA extracted from purified rAAV products as described in Figure 5A, with one probe targeting the *EGFP* gene, the other probe targeting *Bxb1*. A plasmid carrying both *EGFP* and *Bxb1* (pEGFP.Bxb1) was used as the positive control. **B**, Duplex ddPCR was performed with the rAAV products as described in Figure 4J, with one probe targeting the *EGFP* gene, the other probe targeting *PuroR*. Two plasmids carrying *EGFP* and *PuroR,* respectively, were mixed at 1:1 molar ratio to serve as the positive control (pEGFP+pPuroR). n.d.: not detectable.
