# Supplementary figures and images for: High-purity AAV vector production utilizing recombination-dependent minicircle formation and genetic coupling
Source: EMBO Mol Med. 2025 May 16;17(6):1475–94. doi: 10.1038/s44321-025-00248-w (PMC12162853; doi:10.1038/s44321-025-00248-w)

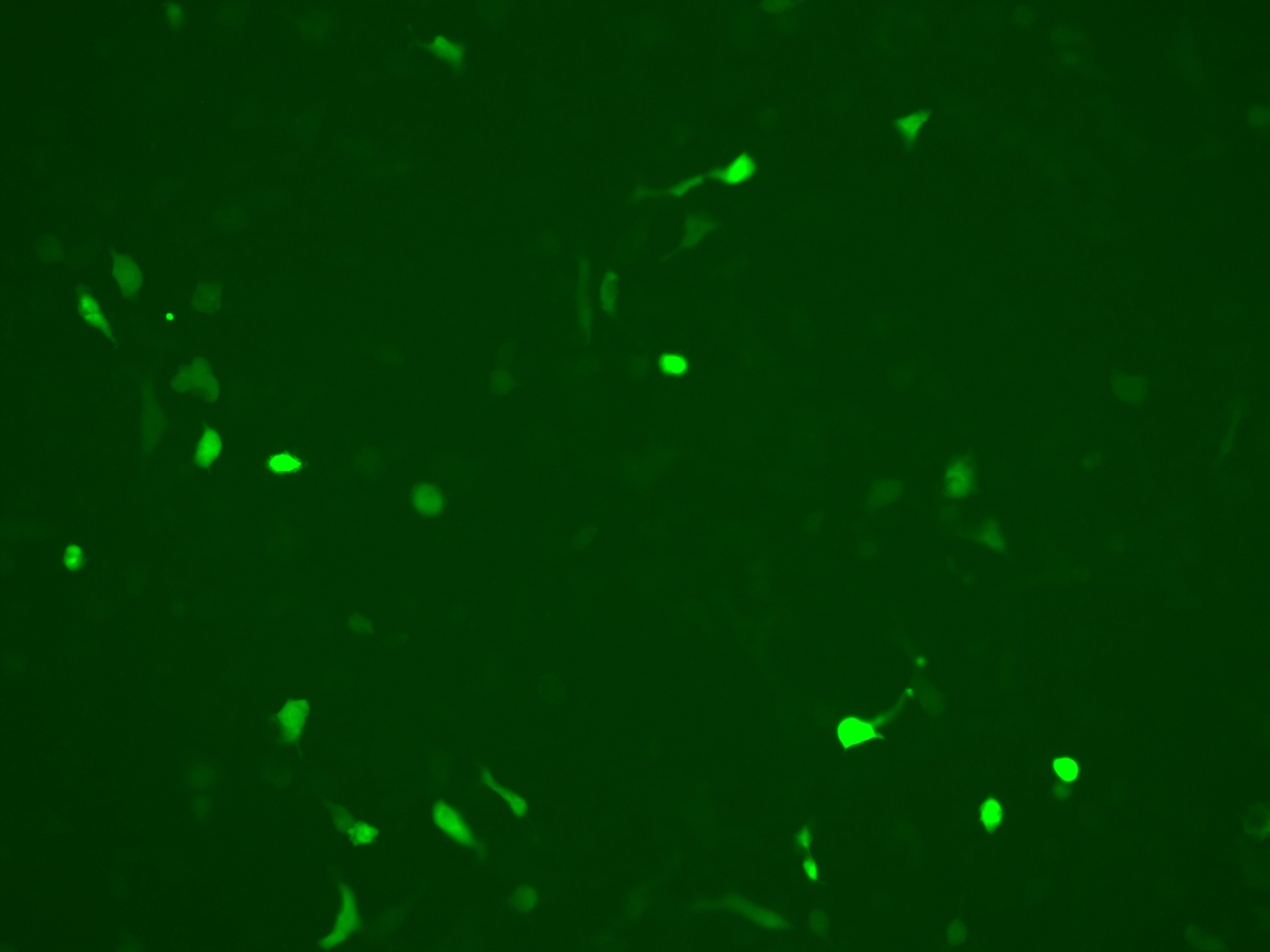

Supplement: Supplementary file 8 — Source data Fig. 2 [file 44321_2025_248_MOESM8_ESM.zip › Figure 2/2D/AAVPureMfg 1.1.jpg]

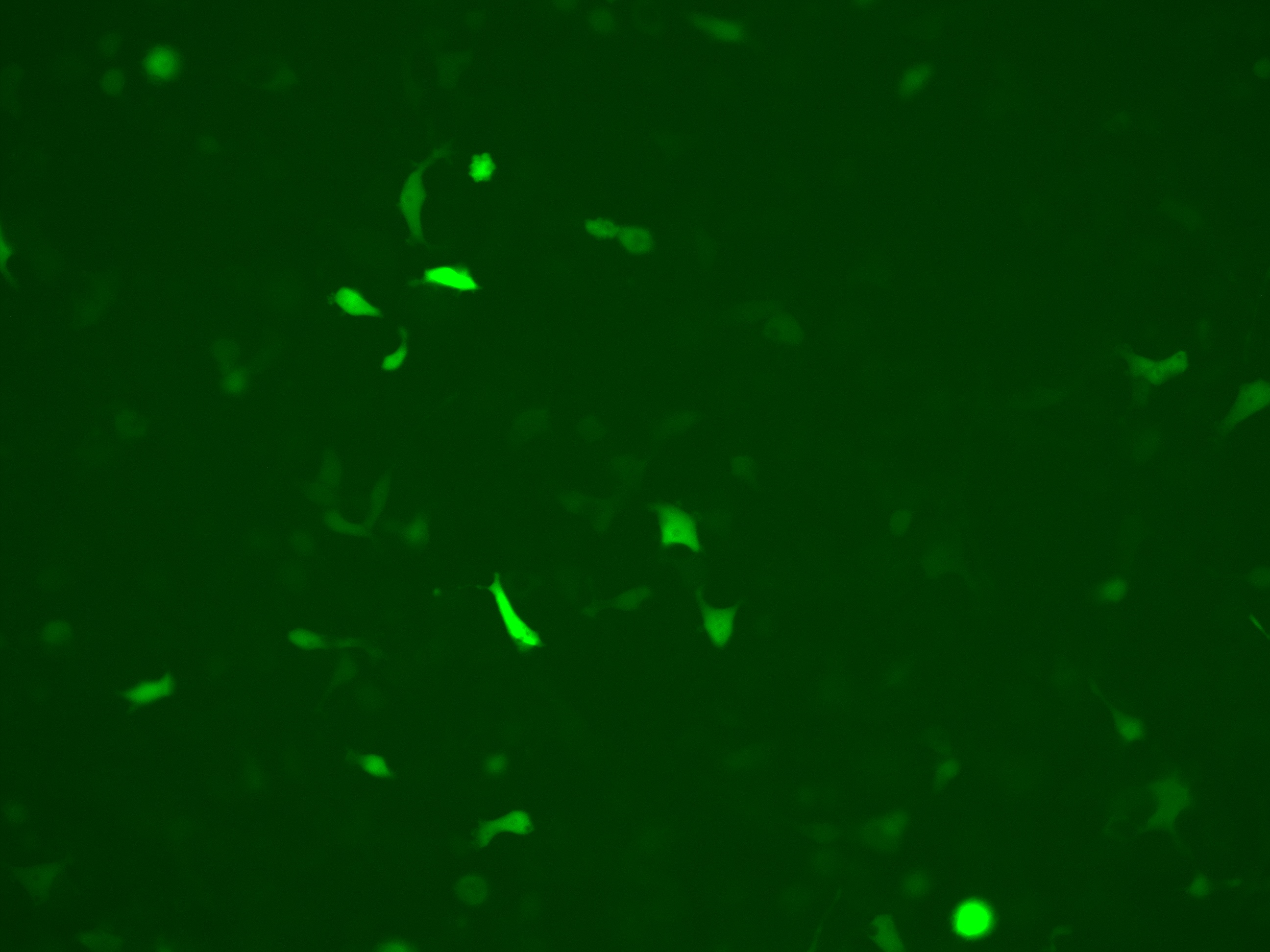

Supplement: Supplementary file 8 — Source data Fig. 2 [file 44321_2025_248_MOESM8_ESM.zip › Figure 2/2D/AAVPureMfg 1.0.jpg]

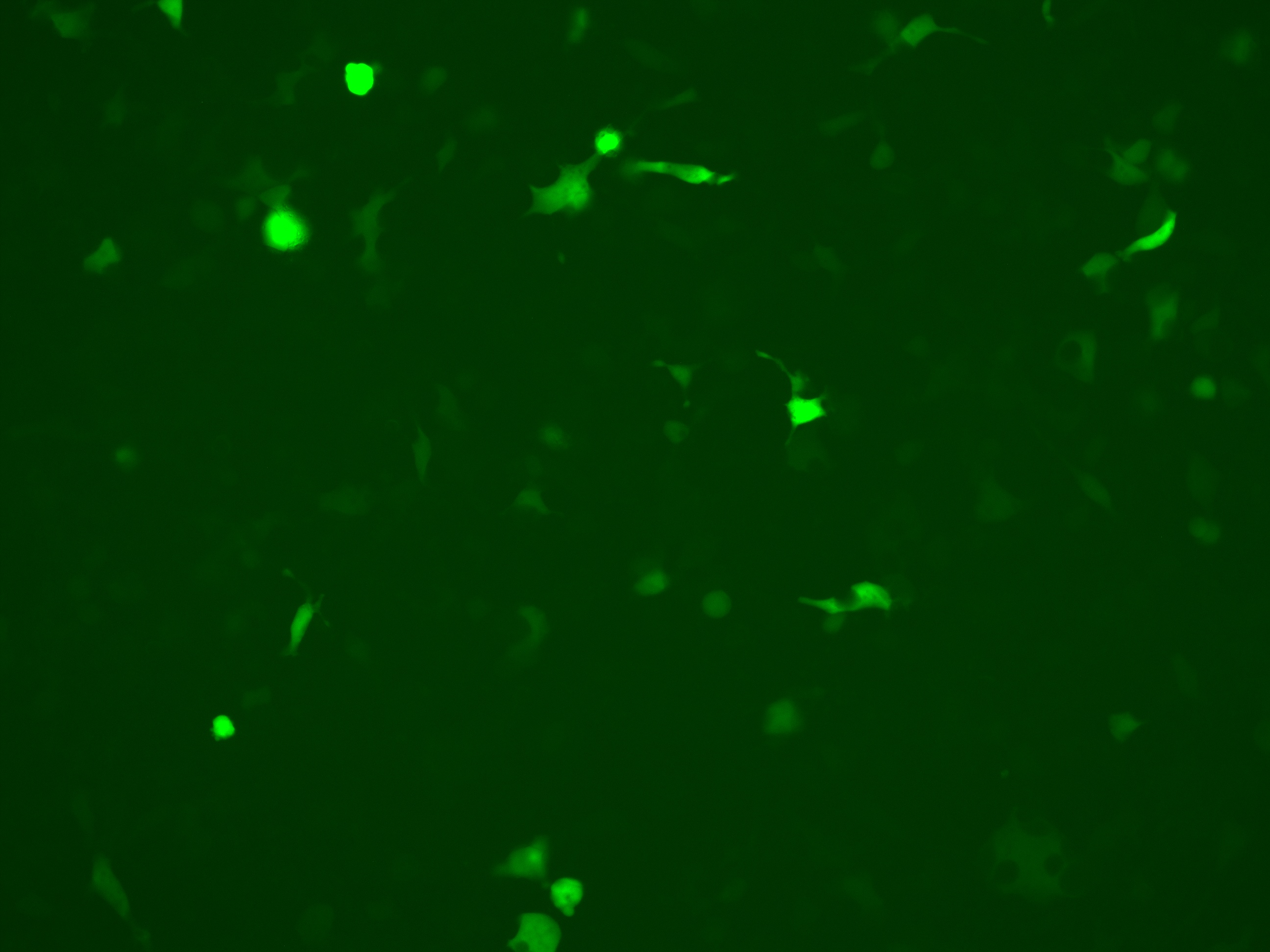

Supplement: Supplementary file 8 — Source data Fig. 2 [file 44321_2025_248_MOESM8_ESM.zip › Figure 2/2D/Triple transfection.jpg]

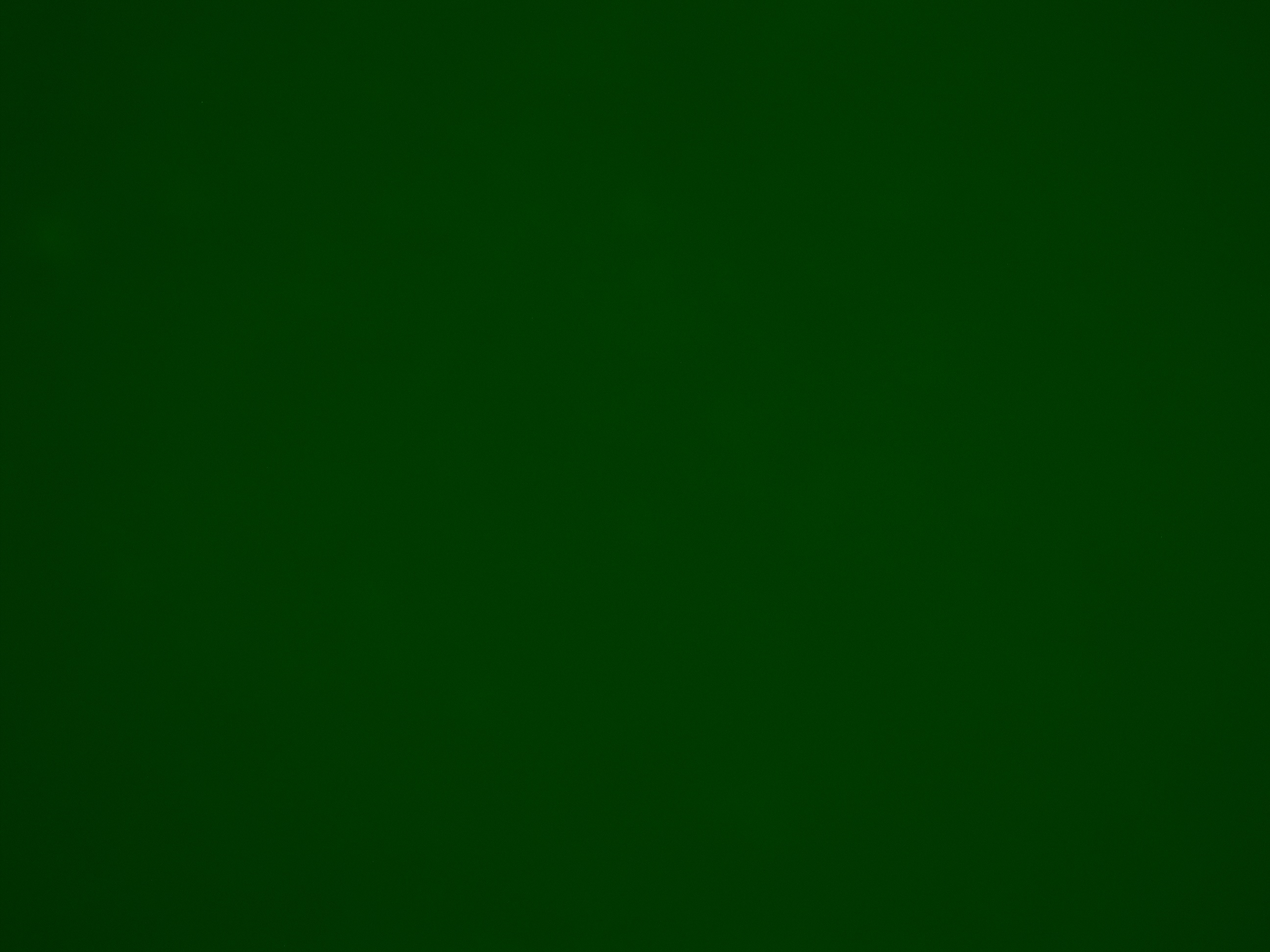

Supplement: Supplementary file 8 — Source data Fig. 2 [file 44321_2025_248_MOESM8_ESM.zip › Figure 2/2D/No transfection.jpg]

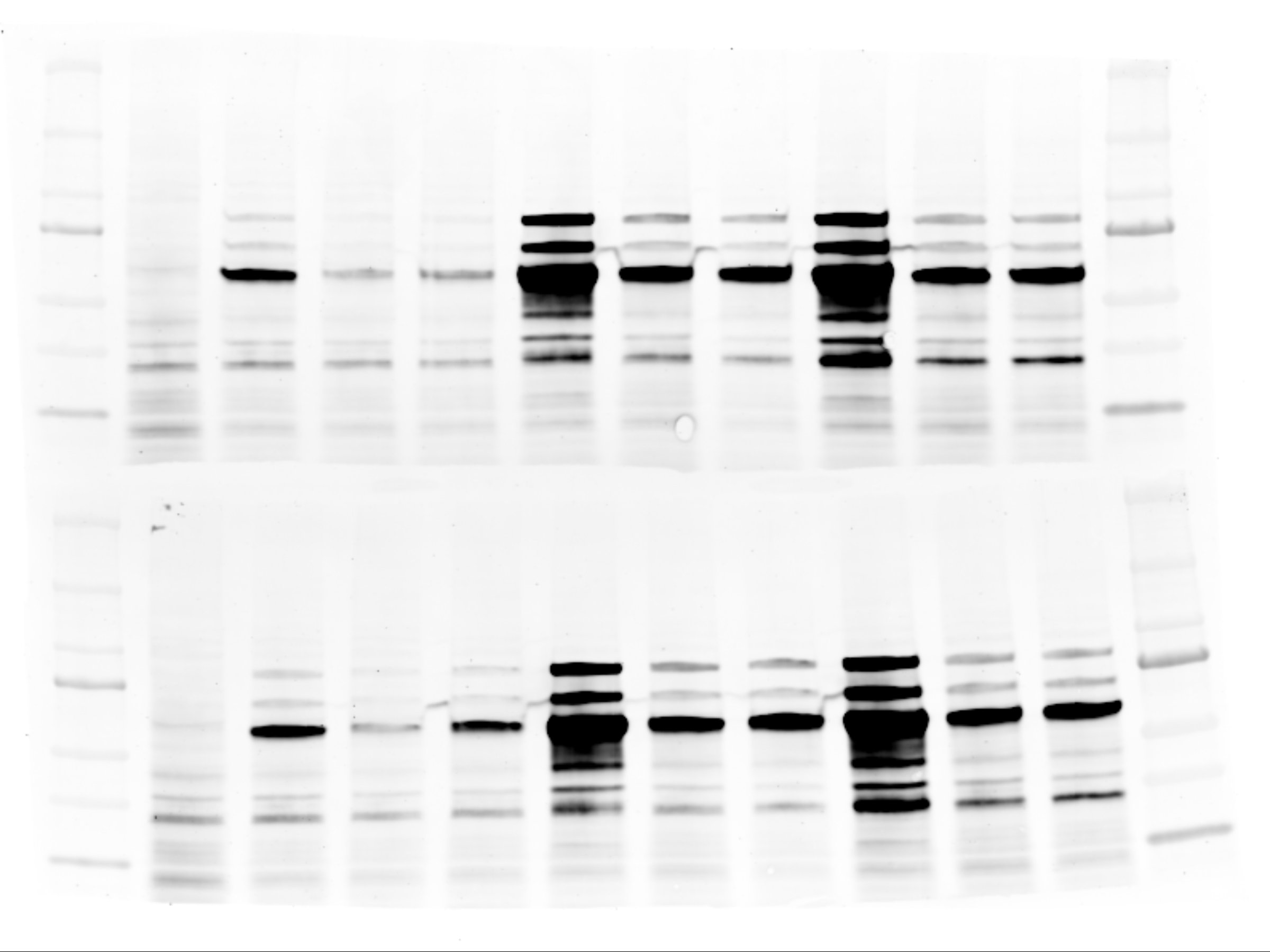

Supplement: Supplementary file 9 — Source data Fig. 3 [file 44321_2025_248_MOESM9_ESM.zip › Figure 3/3E/VP_original image.jpg]

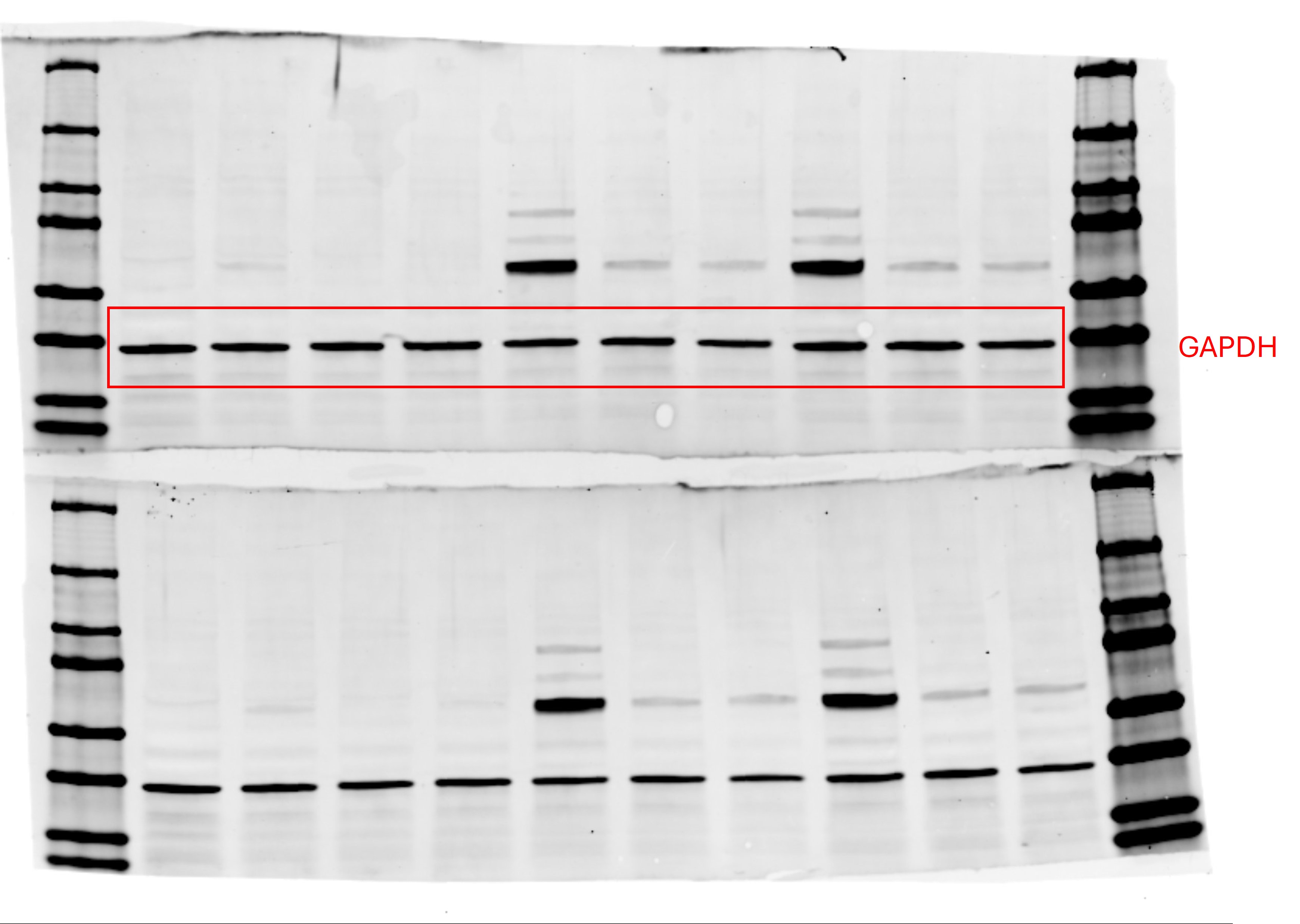

Supplement: Supplementary file 9 — Source data Fig. 3 [file 44321_2025_248_MOESM9_ESM.zip › Figure 3/3E/GAPDH_annotated.jpg]

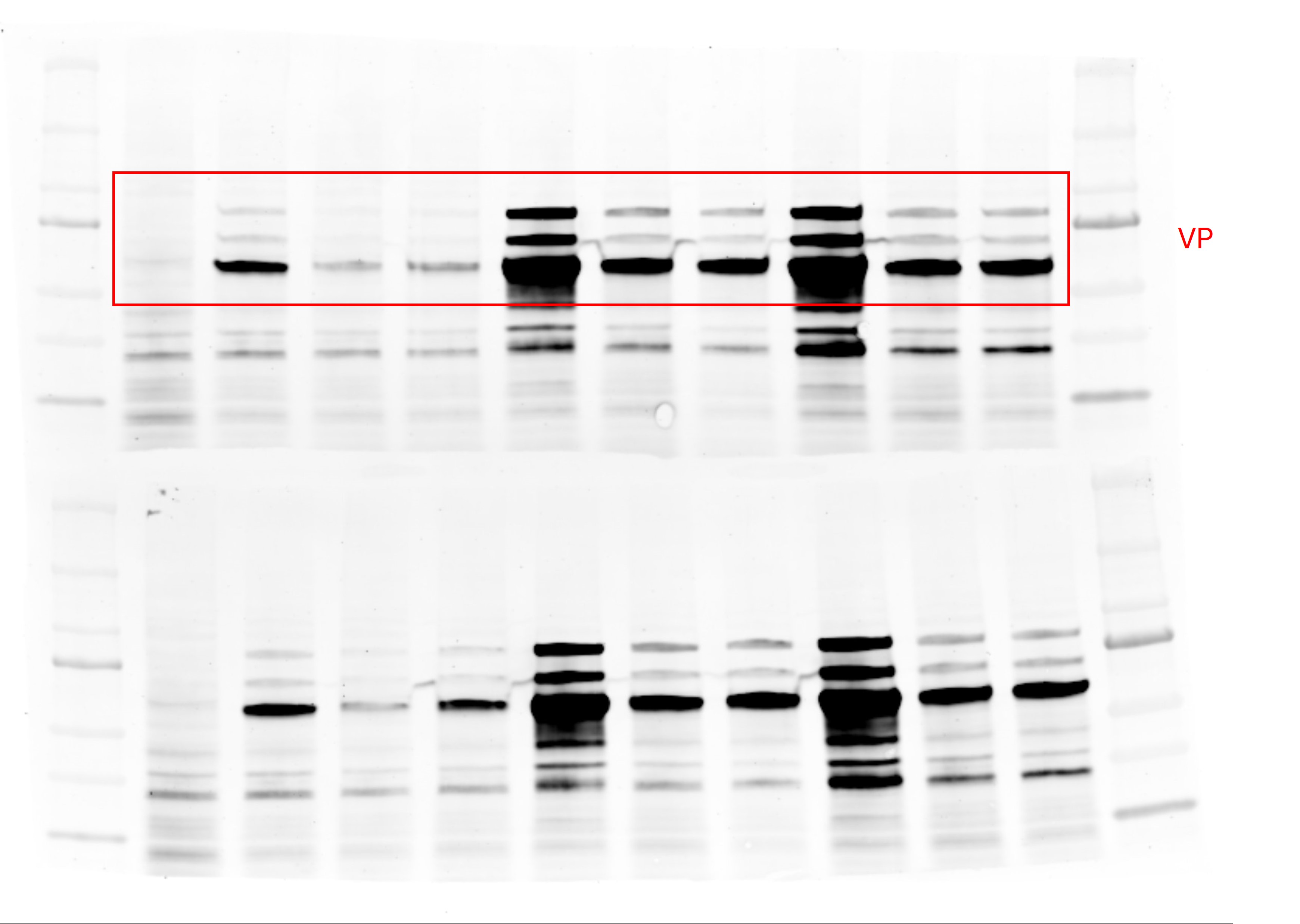

Supplement: Supplementary file 9 — Source data Fig. 3 [file 44321_2025_248_MOESM9_ESM.zip › Figure 3/3E/VP_annotated.jpg]

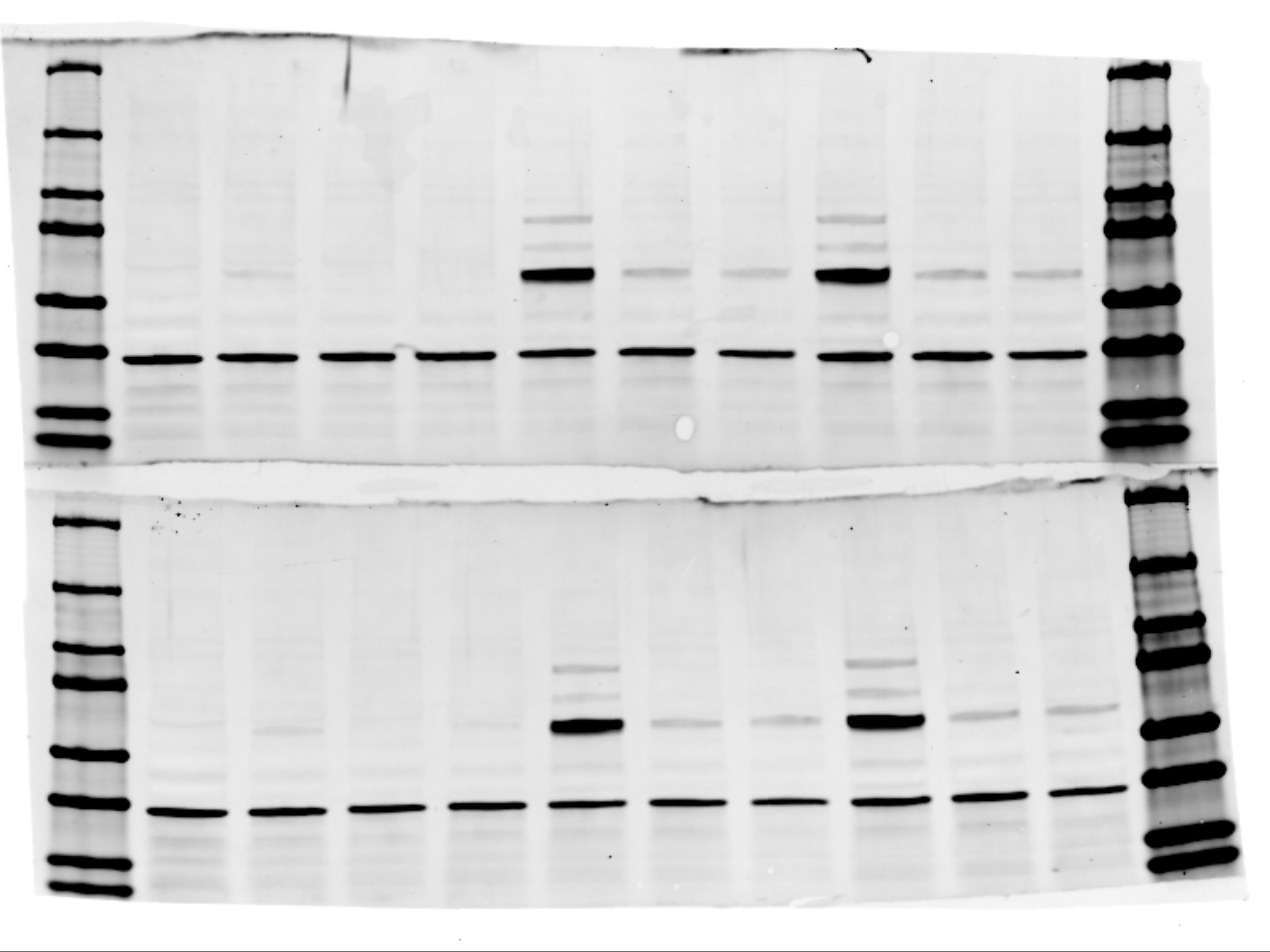

Supplement: Supplementary file 9 — Source data Fig. 3 [file 44321_2025_248_MOESM9_ESM.zip › Figure 3/3E/GAPDH_original image.jpg]

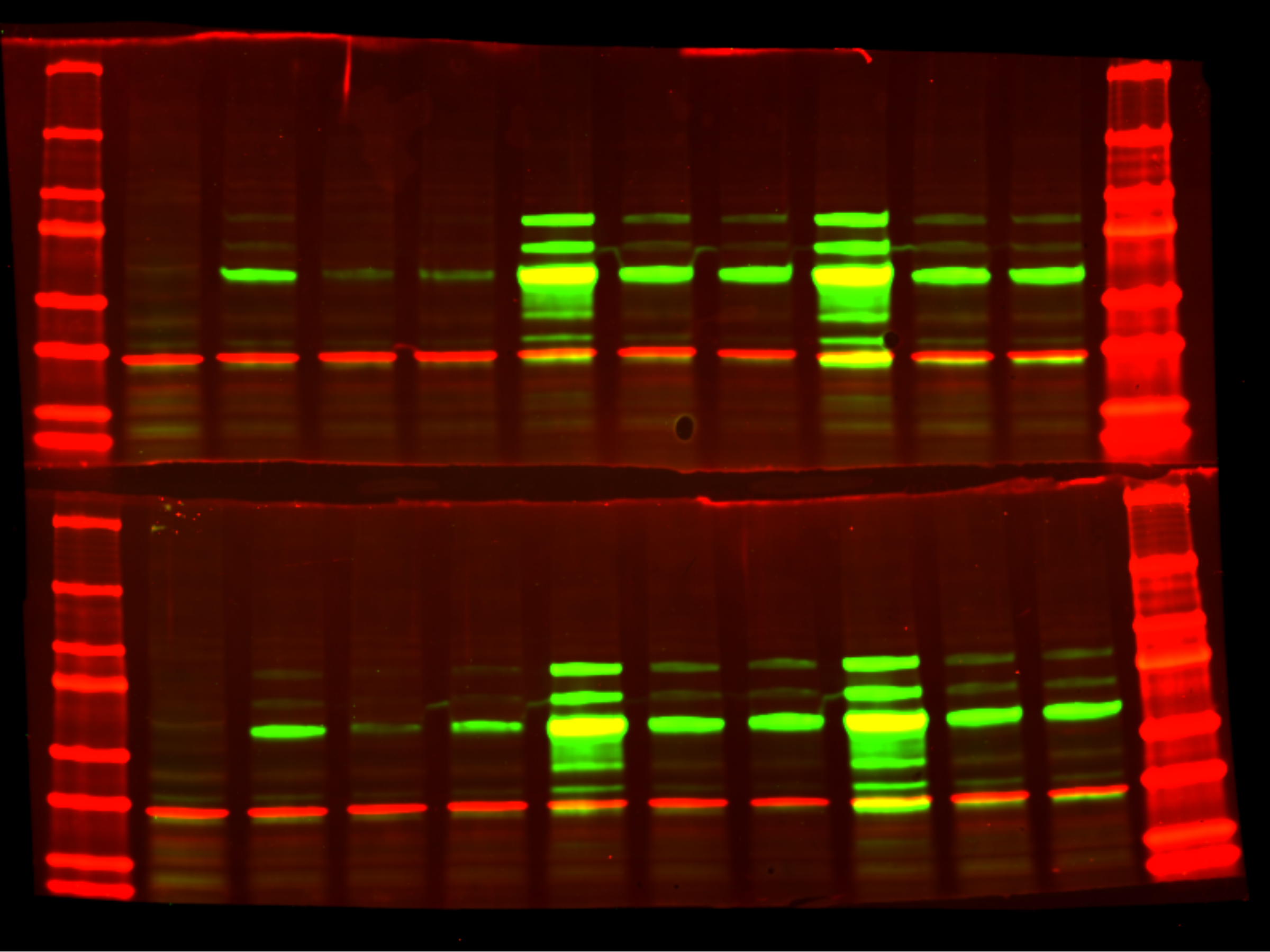

Supplement: Supplementary file 9 — Source data Fig. 3 [file 44321_2025_248_MOESM9_ESM.zip › Figure 3/3E/VP and GAPDH_original image.tif]

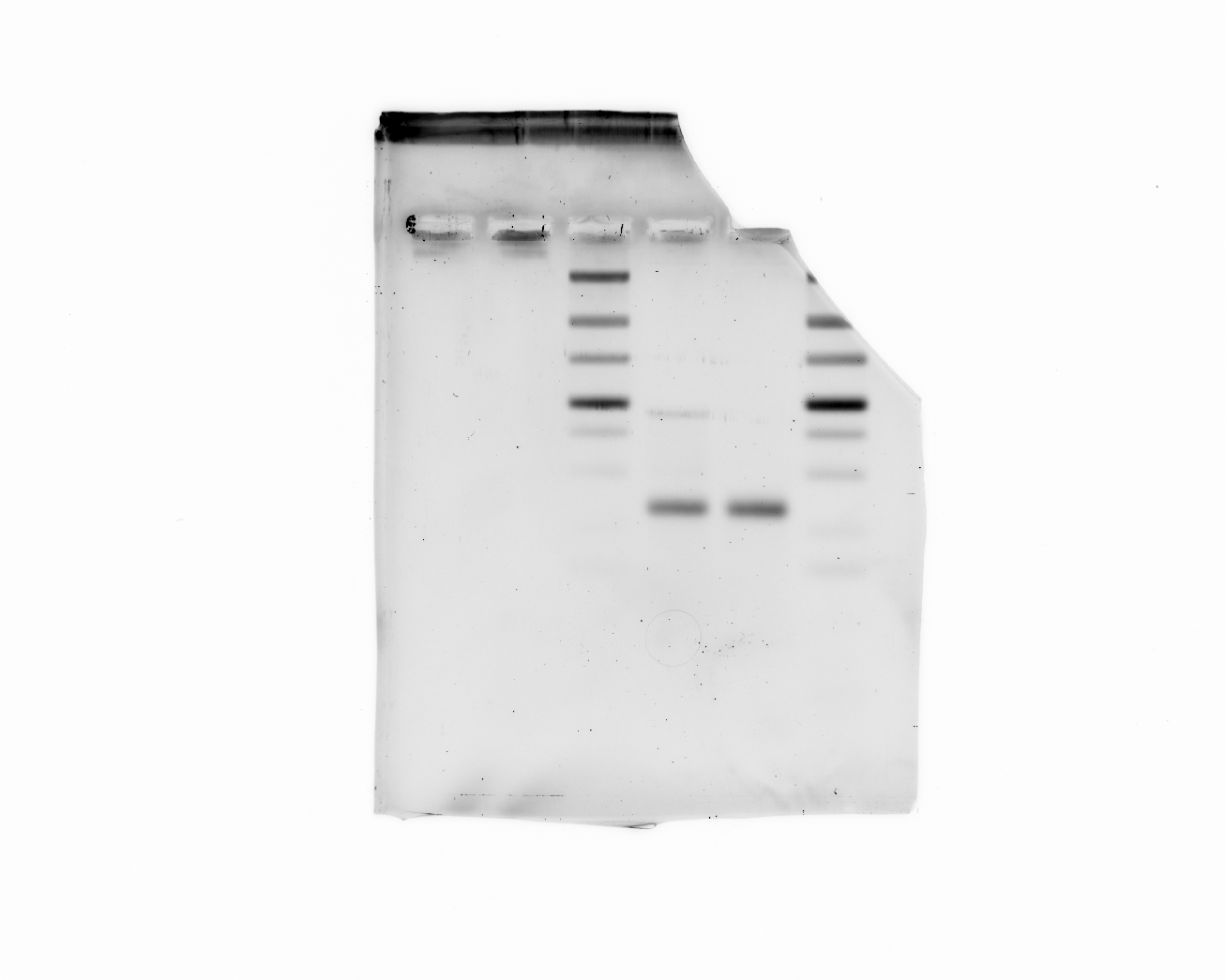

Supplement: Supplementary file 11 — Source data Fig. 5 [file 44321_2025_248_MOESM11_ESM.zip › Figure 5/5E/Alkaline gel.tif]
