# Supplementary material for: High-purity AAV vector production utilizing recombination-dependent minicircle formation and genetic coupling
Source: EMBO Mol Med. 2025 May 16;17(6):1475–94. doi: 10.1038/s44321-025-00248-w (PMC12162853; doi:10.1038/s44321-025-00248-w)
Supplement: Supplementary file 12 — Expanded View Figures [file 44321_2025_248_MOESM12_ESM.pdf]

Expanded View Figures

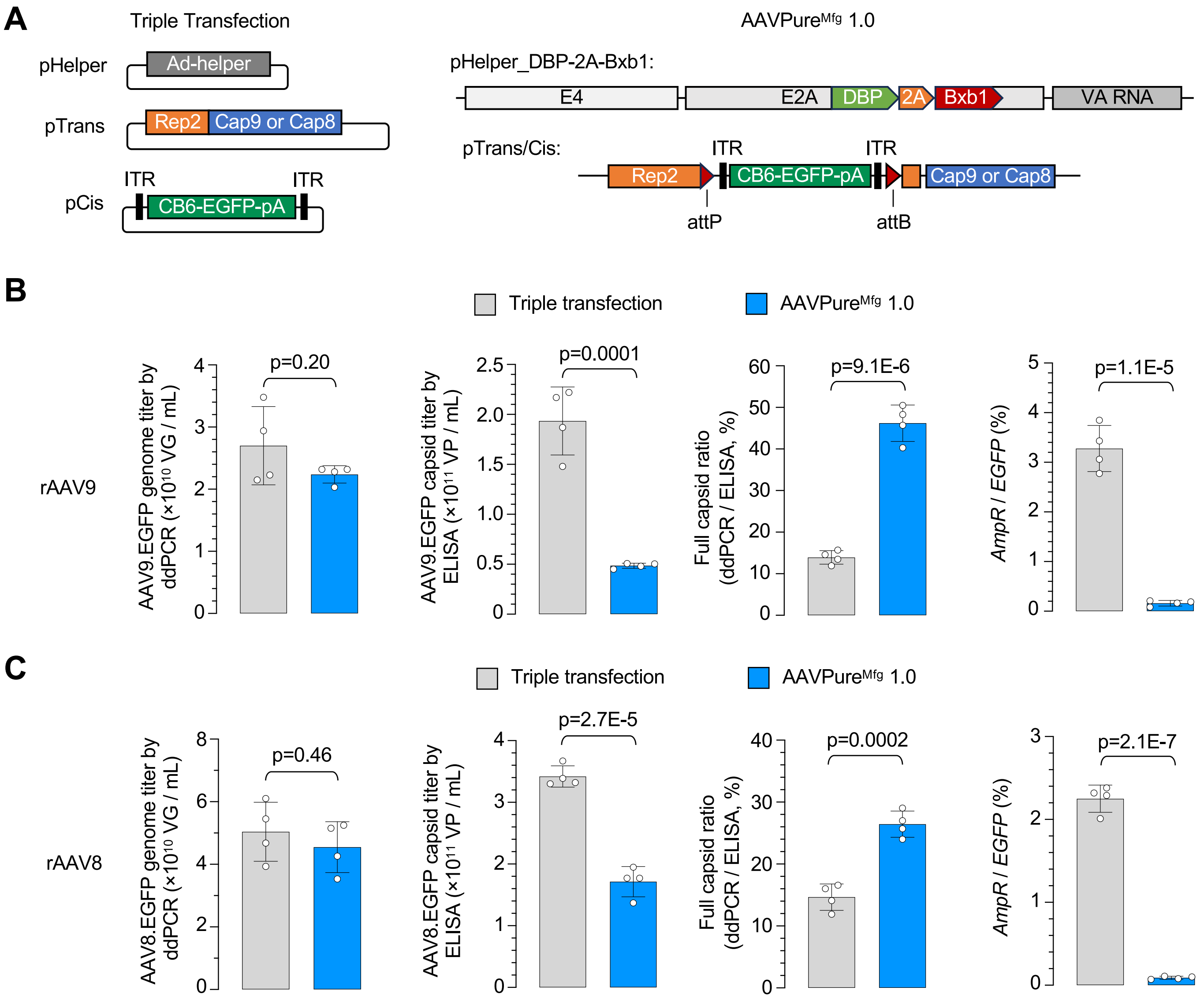

Figure EV1. Application of AAVPure^{Mfg} 1.0 in different AAV serotypes.

(**A**) Schematics of plasmid components in triple transfection (left panel) and AAVPure^{Mfg} 1.0 (right panel) used to produce AAV9.EGFP and AAV8.EGFP vectors. (**B**) Comparison of AAV9.EGFP produced by either triple transfection or AAVPure^{Mfg} 1.0. (**C**) Comparison of AAV8.EGFP produced by either triple transfection or AAVPure^{Mfg} 1.0. The procedure of rAAV production and vector characterization in (**B**, **C**) were the same as shown in Fig. 2. In (**B**, **C**), data are mean ± s.d. of biological replicates, $n = 4$ for each group. Statistical analysis was performed using unpaired *t* test. Exact *P* value was indicated in the figure.

 © The Author(s)

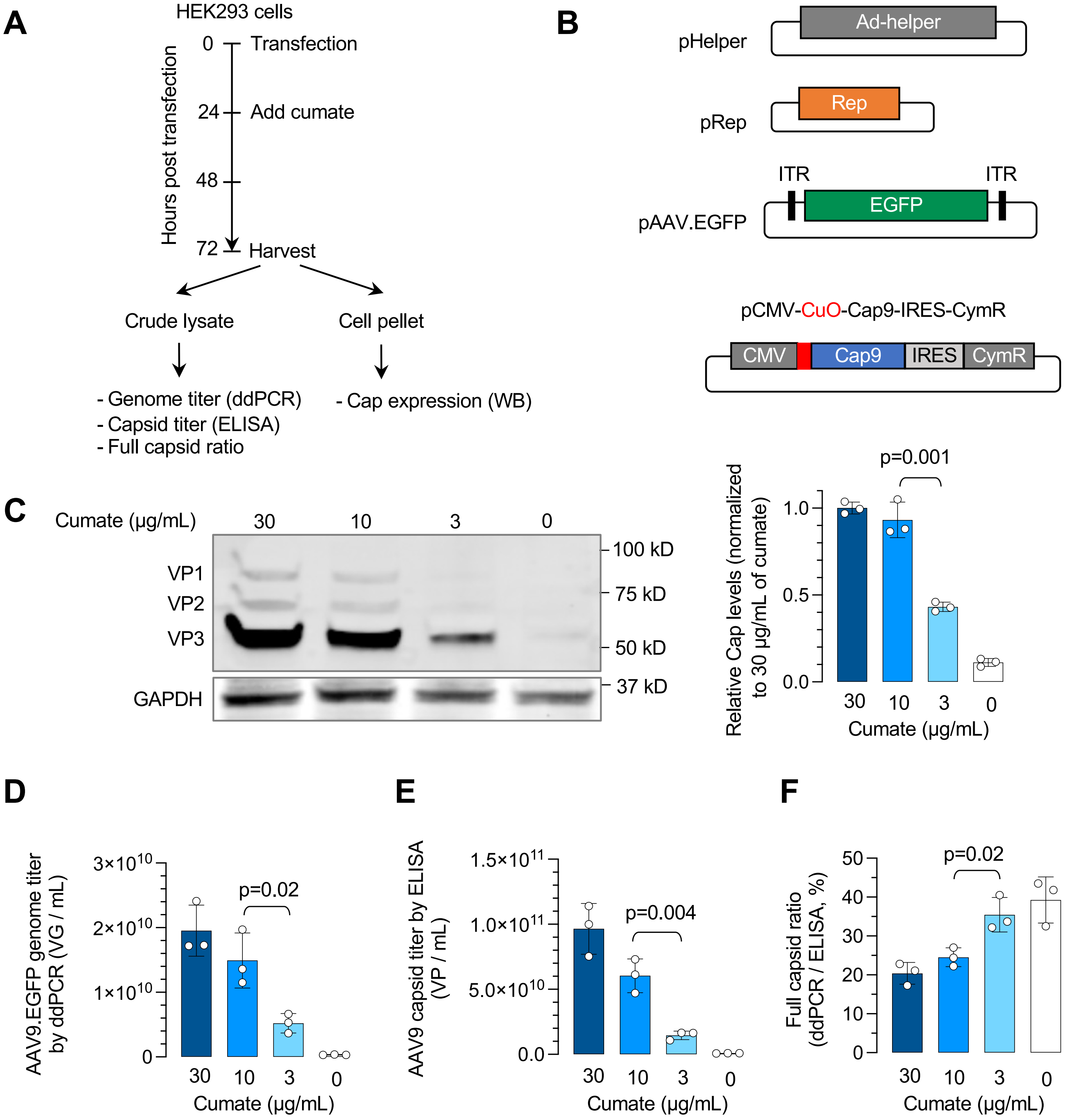

Figure EV2. Lower VP expression increased the full capsid ratio with a marked decrease in vector genome titer.

(**A**) Schematic showing the experimental procedure. (**B**) Construct illustrations of the plasmids used in quadruple transfection to produce AAV9.EGFP. (**C**) Representative western blotting images (left) and the quantification (right) of viral proteins (VP1, 2, 3) at indicated cumate concentrations. (**D–F**) Comparison of rAAV genome titer, capsid titer, and full capsid ratio in cleared lysates with different cumate concentrations. In (**C–F**), data are mean ± s.d. of biological replicates, $n = 3$ for each group. Statistical analysis was performed using unpaired t test. Exact P value was indicated in the figure.

© The Author(s)

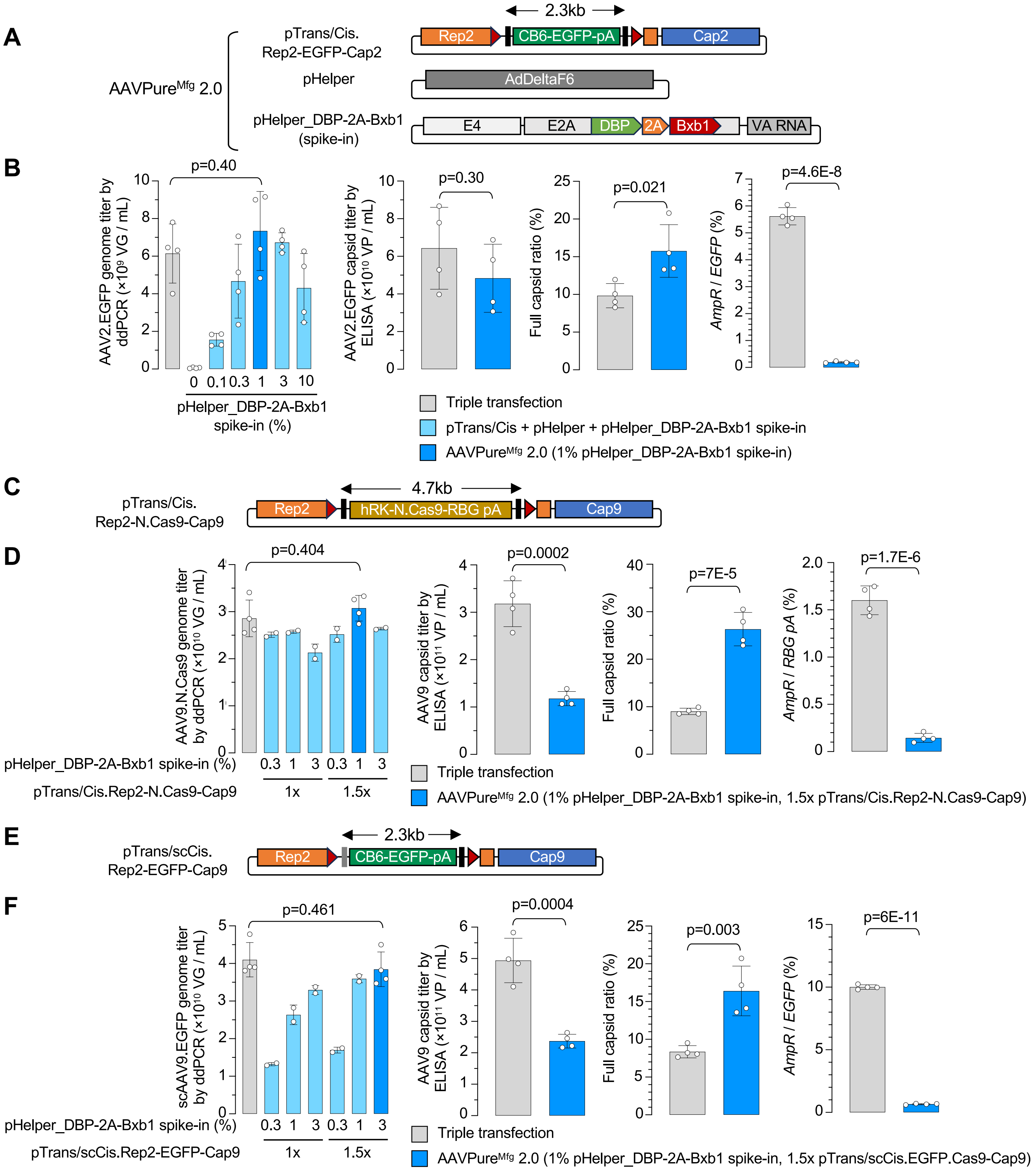

Figure EV3. Application of AAVPure[Mfg] 2.0 in producing AAV vectors with different serotypes, transgenes, and ITR configurations.

(**A**, **C**, **E**) Schematics of plasmid components in AAVPure[Mfg] 2.0 in producing ssAAV2.EGFP, ssAAV9.N.Cas9, and scAAV9.EGFP. In (**E**), the gray bar indicates mutant ITR that results in self-complementary (sc) vector genome. (**B**, **D**, **F**) Comparison of rAAV genome titer, capsid titer, full capsid ratio, and plasmid backbone DNA levels in cleared lysates between triple transfection and AAVPure[Mfg] 2.0. In (**B**, **D**, **F**), data are mean ± s.d. of biological replicates, $n = 4$ for each group. Statistical analysis was performed using unpaired t test. Exact P value was indicated in the figure.

 © The Author(s)

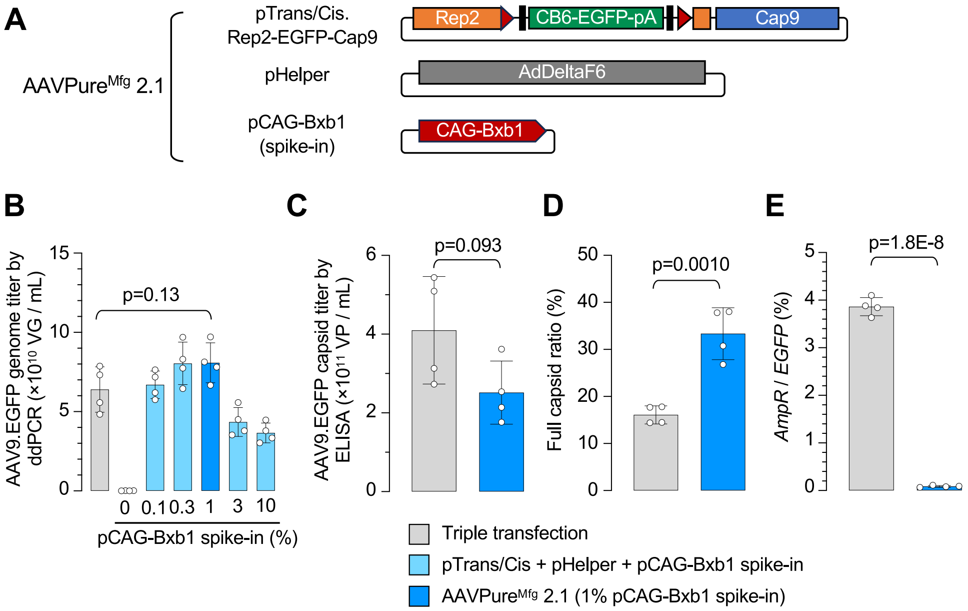

Figure EV4. Developing AAVPure^{Mfg} 2.1 with pCAG-Bxb1 spike-in.

(**A**) Schematics of plasmid components in AAVPure^{Mfg} 2.1. (**B**) AAV9.EGFP genome titer produced by triple transfection or AAVPure^{Mfg} 2.1 with different spike-in amount of pCAG-Bxb1. The rAAV production and titering procedures were the same as Fig. 2B. (**C–E**) Comparison of AAV9 capsid titer (**C**), full capsid ratio (**D**), and plasmid backbone DNA levels (**E**) in cleared lysates between triple transfection and AAVPure^{Mfg} 2.1 with 1% pCAG-Bxb1 spike-in. The detailed plasmid usage is described in Table EV1. In (**B–E**), data are mean ± s.d. of biological replicates, $n = 4$ for each group. Statistical analysis was performed using unpaired *t* test (**C–E**) or one-way ANOVA followed by Dunnett's multiple comparisons test against the triple transfection group (**B**). Exact *P* value was indicated in the figure.

© The Author(s)

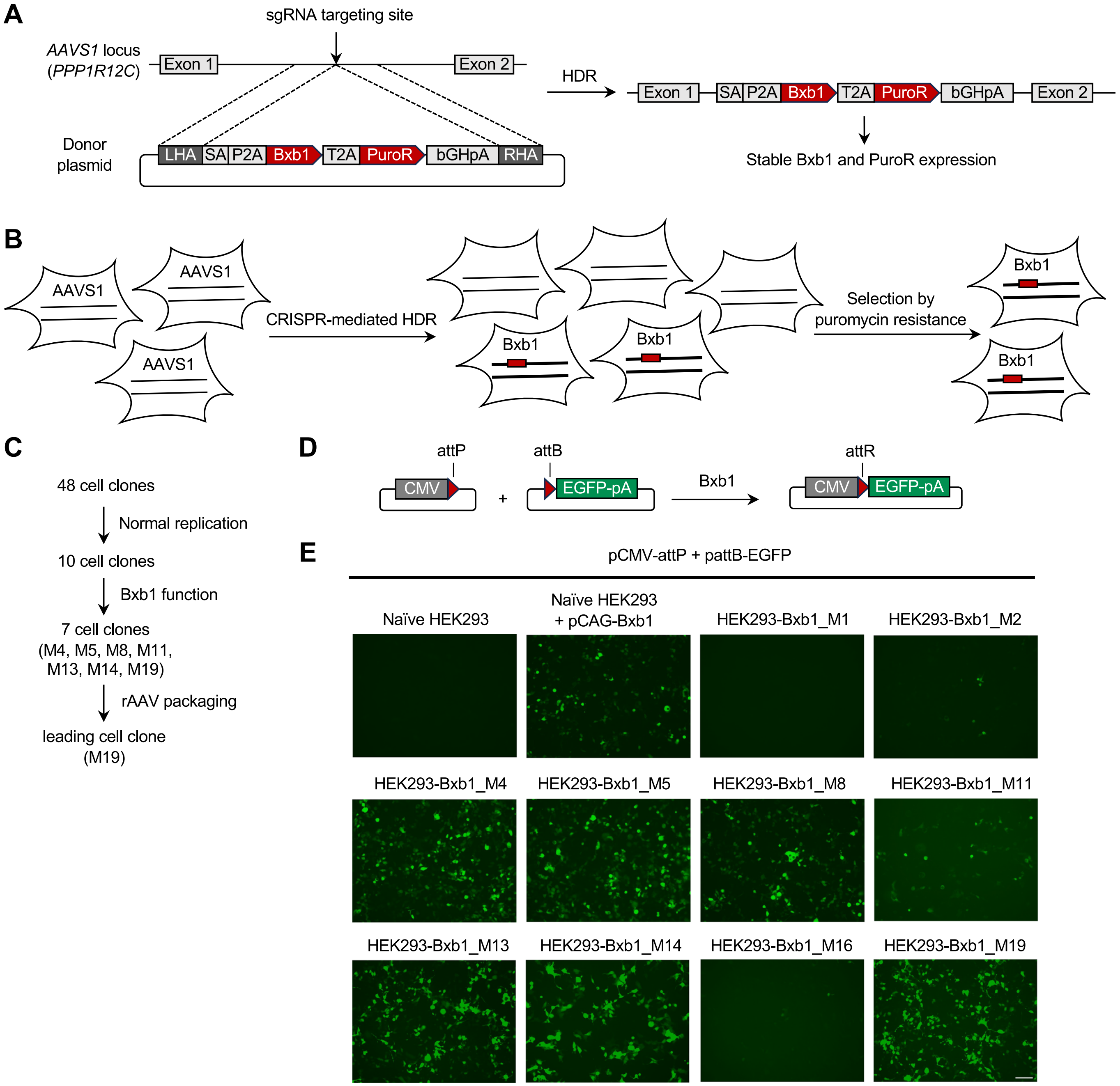

Figure EV5. Generating a monoclonal HEK293-Bxb1 cell line.

(**A**) Schematic diagram illustrating *Bxb1* knock-in into the *AAVS1* site in HEK293 cells. (**B**) Schematic diagram showing the workflow to engineer and select HEK293-Bxb1 cells. (**C**) Procedure of HEK293-Bxb1 monoclonal cell line generation and screening. (**D**) Schematics of a reporter assay to determine Bxb1 recombination activity. (**E**) Representative fluorescence images showing naive HEK293 cells or HEK293-Bxb1 monoclonal cell lines that were transfected with the reporter plasmids, pCMV-attP and pattB-EGFP. Images were taken 1 day post transfection. Scale bar, 100 μm.

 © The Author(s)
